# Modeling and Analyzing the Impact of the Internet of Things-Based Industry 4.0 on Circular Economy Practices for Sustainable Development: Evidence From the Food Processing Industry of China

**DOI:** 10.3389/fpsyg.2022.866361

**Published:** 2022-04-25

**Authors:** Xiaoli Sun, Xuan Wang

**Affiliations:** ^1^Systems and Industrial Engineering Technology Research Center, Zhongyuan University of Technology, Zhengzhou, China; ^2^Department of Management Science, College of Management, Shenzhen University, Shenzhen, China

**Keywords:** Internet of Things, Industry 4.0, sustainable business practices, circular economy, environmental sustainability

## Abstract

The Industry 4.0 concept proposes that new cutting-edge technologies, such as the Internet of Things (IoT), will grow. The acceptance of IoT in the circular economy (CE) is still in its infancy, despite its enormous potential. In the face of growing environmental affairs, IoT based Industry 4.0 technologies are altering CE practices and existing business models, according to the World Economic Forum. This research investigates the function of IoT-based Industry 4.0 in circular CE practices, as well as their impact on economic and environmental performance, which in turn influences overall organizational performance. China-based enterprises provide information for the study, which includes data from 300 companies. Utilizing a structural equation modeling framework known as partial least squares structural equation modeling (PLS-SEM). The major findings are presented in the study: (I) the IoT significantly improves the activities of the CE; (II) the IoT significantly improves the practices of the CE; and (III) the IoT meaningfully advances the practices of CE (green manufacturing, circular design, remanufacturing, and recycling). Moreover, the findings shows that environmentally friendly business practices help enhance environmental performance of firm, while also stimulating their economic performance; and improved environmental performance has a significant positive influence on firm performance. This research lays the groundwork for contributing nations/companies to attain economic and long-term sustainability goals at the same time by incorporating IoT-based Industry 4.0 technology into CE practices.

## Introduction

As the globe becomes more unstable, the markets face more difficulties on a daily basis. The stakeholders’ ability to effectively manage their business is hampered when there is a high degree of indecision in the industry. As a result, the significance of ethical business practices (EBP) has been highlighted (Keshri et al., 2020; Yu, 2022). The organization can grow with minimal risks if the kind of business is blatant and clear. IoT technologies and circular economy (CE) are also encouraging businesses to become more environmentally friendly and efficient (Mohammadzadeh et al., 2018). As a result, businesses are incorporating CE and IoT into their operations to support EBP.

There is a slew of recent reviews in the literature examining the role that digital technologies (DTs) play in advancing CE (Sattarian et al., 2019). Industry 4.0 technologies, such as cyber-physical structures, Internet of Things (IoT), radio-frequency identification (RFID), cloud manufacturing, and stabilizing manufacturing have all been examined in these studies as well as architectural layers involved in data gathering, integrating, and analyzing (Astill et al., 2019). This research has directed the growth of appropriate application settings for DTs in the context of CE’s lifecycle management, digital transformations, supply chain management, and so on (Aggarwal et al., 2021). To name just a few, there have been numerous studies into product-service structures that focus on things like intelligent ways of making and reusing products and services as well as how to keep them in good working order and how to recycle them.

The CE has received increasing attention recently from academics, stakeholders, policymakers, and so on; though the body of information on this matter is still in its infancy and even the literature does not have a complete definition for the CE (Lezoche et al., 2020). For the time being, the CE is most commonly defined as “a system restorative and regenerative by design” by the Ellen MacArthur Foundation, which purposes to maintain components, products, and materials at their maximum usefulness and value at all times (Kurniawan et al., 2021). Industrial environmentalism, blue economy, biomimicry, and Cradle-to-Cradle are just a few of the other fields of study that support the concept of CE (Müller et al., 2021). Contrary to the linear economy, which typically involves manufacturers using raw materials to make products, selling them to consumers, and then disposing of the waste, the CE takes a more circular approach. Through the use of multiple closed-loop cycles for product reuse, remanufacturing, and recycling, the CE aims at facilitating economic growth while minimizing negative impacts on the environment.

The CE’s theoretical foundations can now be applied more easily to real-world economic initiatives thanks to new technologies like the IoT (Liu and Zhang, 2020). Numerous studies have looked at the effects of IoT and digitalization on the development and implementation of CE in the last few years, with varying results. The Ellen MacArthur Foundation, which has made significant efforts to stimulate the CE, has just released two reports on the subject.

More than 14 trillion dollars will be generated by the IoT by 2024, according to the authors (Kawaguchi, 2019; Roy and Roy, 2019; Lezoche et al., 2020) who predicted that 34 billion devices would be fitted in several areas such as smart grid, city infrastructure, housing/home-based mechanization and carriage, industrial methods and healthcare (Zafari et al., 2019). The IoT provides devices with sensors that allow them to communicate and contribute to information set-ups (Yu et al., 2016). With IoT, even stand-alone products can be smart and linked, and materials can be tracked. IoT also helps collect and manage waste from end-of-life products (Ahamad et al., 2021). Reuse, remanufacturing, and recycling are all made possible as a result. Firms and organizations would be able to amass enormous amounts of facts in a brief period of time using this new technology. Because of its ability to track and monitor product activity, IoT is a valuable resource for manufacturers who can use it to improve technical support (Supardianto et al., 2019). The IoT has the capacity to improve renovation and end-of-life processes. It is critical for any business to keep tabs on the progress of its products, say (Pal and Yasar, 2020). Consequently, the IoT could be a valuable tool for companies to monitor their product’s status, usage, and position in real-time throughout their product lifecycle. As a result, manufacturing executives can learn more about how their customers use and implement their products, allowing for a more personal relationship. As a result, manufacturers and their customers will have more fruitful interactions.

The IoT can greatly benefit from integrating with other fields, such as computer engineering. Limited resources can be used for longer periods, assets can be used more effectively, and natural capital can be regenerated for consumption with greater effectiveness and efficiency when CE is implemented. It has also been found in the literature that the IoT is an excellent tool for promoting the use of circular plans and business models in businesses and organizations (Elavarasan et al., 2021). Using IoT to track a product’s status and condition, as well as how it is being used and where it is, is an effective way to implement a circular strategy based on increasing usage (Stich et al., 2020). In the economic cycle, the IoT can act as an assisting enabler by properly organizing previous knowledge about the circumstances, sites, functioning, and quality over time of assets. A number of CE innovators have found practical solutions to resource-related issues using this technology (Zhao et al., 2019). As an outcome, CE models may be useful in extracting value from the enormous amounts of data generated by the IoT. To put it simply, the IoT refers to a wide range of technologies that allow various devices to be connected and monitored *via* a network of data. Physical objects like actuators, smart machines, sensors, and tags can form an IoT network and communicate with each other in order to exchange data and generate new information that will increase the value of the network.

The IoT is built on three pillars: identification, communication, and interaction. When objects are able to sense and communicate with their surroundings, they aid in the understanding of the complexity and the appropriate response (Bhattarai and Kumar Jha, 2019). CE can benefit greatly from IoT, which is one of the most important factors. No matter how much progress is made in DTs, the current linear economy will never be able to address the critical problems with natural resources. Even when the economy is linked to DTS, it offers a podium for re-thinking its schemes and certifying CE. New business models will be able to be scaled more effectively if CE common codes are combined with IoT and cloud technologies.

An evaluation model for the impact of IoT on CE in order to achieve sustainable business practices has been developed in light of this. The seminal articles are used to identify and assess the concepts of sustainable business practices, CE, and IoT. IoT becomes increasingly important as the organization strives to be more environmentally friendly through its use of CE operations. In addition, the guiding principles of a company’s business operations have a substantial influence on the quality of its products and services (Khan et al., 2021a). In addition, a company’s functioning and practices reveal its principled side, which comprises employee involvement, environmental well-being, and customer satisfaction from their products or services (Hopkins, 2021). Thus, the work suggested a CE-IoT model that included elements from CE, and IoT. The model calculates the aspects that impact a company’s decision to adopt CE-IoT. In the above context, the research question is as follows: In a VUCA world, how can we model the adoption intention of CE-IoT in the food processing industry?

In order to find an answer, the variables were chosen from a preexisting literature review and statistically validated using the Churchill approach. In order to assess the food processing industry’s interest in implementing CE-IoT, several relevant factors were considered. The study is notable for a number of reasons. First and foremost, this is inventive work that pools various methods and endorses a placement framework that integrates CE, and IoT factors into a single platform. Herein lies the research’s greatest contribution. This study’s unique contribution is that all of the factors were classified using an Artificial Neural Network (ANN) to ensure its robustness as a mechanism for testing the CE-IoT. Third, the use of ANN-based cataloging in the food processing industry perspective advances the adoption of CE-IoT at the strategic level.

The following is a description of the paper’s organizational structure: section “Theoretical Frameworks and Hypothesis” discusses the theoretical framework as well as the development of hypotheses. Section “Research Methods and Data” discusses data sources as well as research methodologies. Section “Results Analysis and Findings” contains a description of the findings as well as a discussion. Section “Discussion” is comprised of conclusions and managerial ramifications of the findings.

## Theoretical Frameworks and Hypothesis

Many people look at this topic from two different angles when debating the link between environmental stewardship and economic growth. The first step is the win-lose game, in which strategic options with environmental ambitions and real economic implications are weighed against one another. When all parties involved are satisfied, a win–win partnership is achieved. It is futile to assume a zero-sum game and neglect the opportunity to “grow the pie” for all contributors (Chen et al., 2020). Other actions may be more expensive and not be rewarded because of the current economic system, whereas certain environmental sustainability programs pay both participants.

Many investigators and experts from a wide range of disciplines have recently studied how businesses might incorporate environmental challenges into their operations by adopting frameworks such as ecological foot-printing, triple-bottom-line, business ecology, and life cycle management (Li et al., 2020). We may learn from these theoretical frameworks about how to combine ecological, financial, and social problems into our business strategies. They do not fundamentally replace each other, but rather explain various aspects of the same phenomenon. To describe stewardship of the social, economic, and environmental realms, a more comprehensive approach is needed. The study adheres to two consistent theoretical frameworks: ecological modernization theory (EMT) (Bag et al., 2021) and practice-based perspective theory (PBV) (Rejeb et al., 2020).

Economic expansion, according to the EMT, has led to environmental concerns, which can be alleviated by increasing resource efficiency through technological innovation, such as green supply chain techniques. It is in this context that environmental protection is no longer a “problem,” but rather an “opportunity,” which supports concepts like “ecologizing economy” (Tiwari and Khan, 2020) and “economizing ecology” (Pieroni et al., 2021). With this goal in mind, the Public Benefit Corporation teaches supply chain strategies that are environmentally friendly (Koistinen et al., 2022). Bag et al. (2021) popularized PBV, which is an extended form of the mainstream resource-based view theory (RBV) The adoption of “an established activity or collection of activities that other organizations may execute” (as defined by PBV) causes variances in enterprises’ performance, as explained by PBV (Ntsondé and Aggeri, 2021).

**FIGURE 1:**
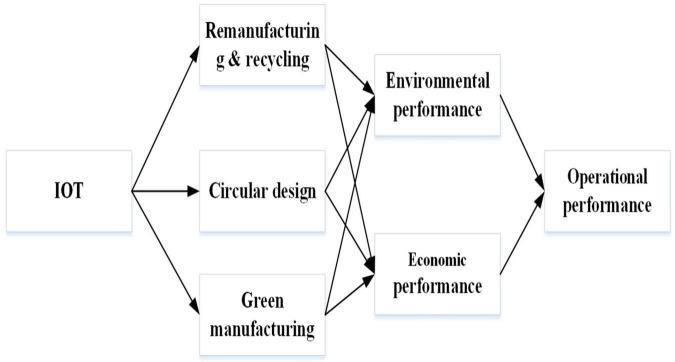
Conceptual framework.

We built an inclusive SEM framework around eco-environmental practices (circular purchasing, recycling, remanufacturing, and circular design) based on the theoretical foundations of environmental transformation and resource-based view, which are driven by IoT in the context of Industry 4.0, and finally lead to entire firm performance. Table 1 explains the definition and constructions of the term. The study’s conceptual framework can be seen in Figure 1.

**TABLE 1:** Definition of constructs.

Constructs	Definitions
Internet of Things (IoT)	Internet of Things enhances supply chain transparency through more data sharing and smart deals, which supports to development of long-term partnerships with supply chain allies and improves the efficiency of work processes (Appolloni et al., 2022).
Remanufacturing and recycling (RR)	To put it another way, “remanufacturing” keeps a product’s original shape, while “recycling” breaks it down into its constituent parts and then melts, smelts, or processes them into new ones.
Circular design (CP)	By working with suppliers to obtain environmentally friendly materials, CP aims to lessen the environmental impact of its products.
	In addition, environmental performance is a factor in supplier selection (Hartley et al., 2020; Rehman Khan et al., 2021).
Circular design (CD)	Companies can decrease waste through recycling, remanufacturing, and refurbishment thanks to reverse logistics. It also allows environmentally friendly purchasing and manufacturing (Jinru et al., 2021).
Environmental performance (ENP)	A company’s ability to reduce waste and emissions is reflected in this metric. Toxic/harmful chemicals and materials can be minimized in the supply chain by using this method (Alkhuzaim et al., 2021).
Economic performance (ECO)	For example, it demonstrates the company’s ability to reduce production process expenses such as material acquisition and remanufacturing processes, reusing and recycling materials as well as energy and water use (Hull et al., 2021).
Operational performance (OP)	In comparison to the industry average, this metric measures how well the company performs economically, financially, and marketing-wise (de Morais et al., 2021).

### Industry 4.0 and the Circular Economy

Ecological economics emphasizes the concept of product manufacturing, use, and recycling in order to create sustainable development for future generations at the micro and macro levels. Accordingly, today’s industries are focused on a circular operating model instead of linear economic models in order to make the process more efficient and sustainable. These kinds of conditions necessitate an approach that prioritizes zero waste, resulting in improved usage of materials, energy, and scarce resources (Grafström and Aasma, 2021). CE is a common term for this occurrence. Manufacturing, construction, electronics, automotive, and apparel industries have seen a rise in the use of computer-aided design (CAD) (Ionescu, 2021; Lăzăroiu et al., 2021; Nica et al., 2021; Vătămănescu et al., 2021). The impact of CE on the food processing industry, on the other hand, is largely unexplored. The CE has also failed to fully benefit several industries as a result of a slower rate of adoption. Everyday challenges like technological, organizational, and regulatory standards are to blame for this (Kumar et al., 2021). Centobelli et al. (2021), on the other hand, looked at several works and emphasized the necessity of creating a comprehensive evaluation framework for CE in the industry. As previously mentioned, Gupta et al. (2021) emphasized the difficulty of evaluating CE at the firm level and the need for a thorough approach to understanding CE implementation. As a result, the study’s focus was on determining whether the food processing industry has any interest in implementing CE procedures.

### Internet of Things and Circular Economy

Key CE enablers include DTs, in particular those associated with Industry 4.0. Many companies can share data in their supply chains and track their products using these technologies (Singhai and Sushil, 2021). They can help these companies retain the value of their products more effectively. To ensure a smooth transition to CE, the IoT is considered an important technology (Cheng and Wang, 2021). The IoT can be used to extend the lifecycle of products, according to a number of researchers. In addition, the literature shows that the IoT has the potential to affect a wide range of CE issues.

The IoT is a new paradigm in which objects can sense and communicate with each other, allowing for new ways of exchanging information between them. IoT’s theoretical potential to support the transition to CE has been highlighted in some recent reports published recently (Thomasian and Adashi, 2021). It raises the profile of assets in a particular industry, which has ramifications for CE. Manufacturers, for example, will have access to information about the current and future conditions of their products. Based on the actual performance and usage, they can then offer certain products or services (Kovacova and Lãzãroiu, 2021; Popescu et al., 2021; Rogers and Katarina, 2021). In addition, if products are connected to the IoT, they can be monitored throughout their lifecycles; this helps companies that use circular business models make better decisions (Quan et al., 2021). Maintenance, reusing, remanufacturing, and recycling are all described as possible outcomes of IoT implementation in the CE sector in the literature. According to Pagoropoulos and his colleagues, the IoT can be used to monitor people’s health and the actions of connected products. Based on these arguments, we propose the following hypotheses:

**H1:** The Internet of Things has a significant and positive effect on green manufacturing (GM).
**H2:** The Internet of Things has a significant and positive effect on recycling and remanufacturing (RR).
**H3:** The Internet of Things has a significant and positive effect on circular design (CD).

### Internet of Things Applications in Circular Economy and Environmental Performance

Resource flow management can be made easier in the transition to a CE by leveraging the IoT technology. In order to connect stakeholders from all points of the value chain, the IoT collects data generated by various sensors, such as smart meters (Khalil et al., 2021; Sulich and Sołoducho-Pelc, 2021; Pervez et al., 2022). Aside from providing real-time data, the IoT shows the impact of specific actions taken by stakeholders (Nica and Stehel, 2021; Novak et al., 2021). It is thus possible to develop and use CE models based on IoT-captured data in order to evaluate particular items throughout their lives, such as smartphones (Zając and Avdiushchenko, 2020). The concept of a smart CE has recently been discussed by scholars and practitioners and is facilitated by essential technologies such as the IoT. There is a smart circular strategy framework for manufacturing organizations leveraging IoT that is utilized to align activities across the CE and information systems (Sarfraz et al., 2020a,b). The IoT’s ability to collect data has led to its widespread use by the CE in the development and implementation of systems (Ding et al., 2020; Kurniawan et al., 2021).

There have been a few examples of how IoT can be used in CE to improve sustainability. For instance, an IoT-based sustainable CE technique from Indonesia for a smart waste management system was introduced by Husgafvel et al. (2022). In order to attain sustainability in smart cities, IoT technology has also been applied in garbage management (Khan et al., 2021b; Kristensen et al., 2021; Su and Urban, 2021; Belhadi et al., 2022). Data-driven decision-making models may be possible because of IoT technology’s data-collecting efficiencies and high levels of data exchange, according to a recent report. An agent-based IoT platform that encourages citizen participation in recycling tasks through gamification mechanisms was proposed by Yalçın and Foxon (2021). In light of the aforementioned research, we have come up with the following hypotheses:

**H4:** Circular design has a positive effect on the environmental performance of the firm.
**H5:** Remanufacturing and recycling has a positive effect on the environmental performance of the firm.
**H6:** Green manufacturing has a positive effect on the environmental performance of the firm.

### Internet of Things Applications in Circular Economy and Economic Performance

It is important to consider the costs and benefits of new technology before jumping on the bandwagon of innovations. In recent policy work, the IoT has been overlooked. There have been studies done in the past to try to figure out how the Internet affects research productivity, the value of the knowledge it provides, and the role it plays in fostering entrepreneurship (Su and Urban, 2021). New technologies can have a significant impact on productivity, which is a crucial driver of economic growth. The IoT is the primary focus of this study (IoT).

To get a sense of how the IoT will affect the economy, it is helpful to go back and review the history of how ICT has influenced economic growth. During the last three decades of research, a period that has seen a revolution in information technology, Yu et al. (2021) famously advocated this topic in the field of economics. There have been two main approaches taken by economists to the problem: micro and macro. If you are interested in how different types of assets affect output or labor productivity, growth accounting is a dynamic technique that looks at how those assets are linked to each other. Data at the country or industry level is typically used to make this determination (Iqbal et al., 2021; Liu K. et al., 2021; Liu Z. et al., 2021; Yumei et al., 2021). Regression analysis may be used to uncover causal effects in econometrics by incorporating heterogeneity and time into the equation. When it comes to measuring the influence of new technology, the econometric approach demands a lot of data over a lengthy period, which might be difficult. As a result, we use the growth accounting method. Based on the findings cited above, we propose the following theories as possible answers:

**H7:** Circular design has a positive effect on the economic performance of the firm.
**H8:** Remanufacturing and recycling has a positive effect on the economic performance of the firm.
**H9:** Green manufacturing has a positive effect on the economic performance of the firm.

### Internet of Things Applications in Circular Economy and Operational Performance

Internet of Things-based I4.0 was originally discussed in the industrial industry in 2011. Several researchers, including myself, provided some initial guidance on how to implement I4.0. However, there is still a need for systematic research into how I4.0 will affect future industries’ operations management. As a result, there are few studies on I4.0’s contributions to operations management that discuss the framework or practical implications of those contributions. All operations management activities could benefit from the automation of procedures brought about by I4.0 technologies (Liu K. et al., 2021; Lv et al., 2021, 2022; Yang et al., 2021). Real-time information on operating units, such as material flow, customer demand, and inventory position of SC echelons, are some examples of applications.

It is possible to share operational resources utilizing cloud manufacturing technology and the IoT. Design, manufacturing, and assembly participants in the supply chain can all benefit from a centralized service platform in the cloud. Another technology made possible by I4.0’s cloud services is additive manufacturing. From a management standpoint, though, I4.0 and sustainability play a vital role in today’s operating system. In conjunction with one other, they have the potential to advance a more environmentally friendly society (Pieroni et al., 2021). Sustainability and the IoT may be able to address environmental and economic concerns in enterprises’ operations management simultaneously. The green design of products and processes, as well as environmentally friendly supply chain operations, play a role in sustainable operations that are ecologically affected. Jinru et al. (2021) have proposed a classification of environmentally sustainable operations that includes (a) design of the environment, (b) cleaner production, and (c) green supply chain management (GSCM). For environmentally friendly operations, Huang et al. (2021) recommended implementing the 3Rs (reduce, reuse, and recycle). As a result, the connections between environmentally sustainable operations and Industry 4.0 are important, given the role that technology plays in making environmentally sustainable operations excellence judgments.

**H10:** Environmental performance has a positive effect on the operational activities of the firm.
**H11:** Economic performance has a positive effect on the operational activities of the firm.

## Research Methods and Data

### Research Methods

Multiple associations between manifest and latent variables were examined simultaneously using a survey and structural equation modeling as a method of multivariate data analysis. In this section, we describe the research instrument design and the sample size, and then we describe the survey process itself. For the data analysis, we used partial least squares structural equation modeling (PLS-SEM) version 6.0 software, which can handle a wide range of both direct and moderated effects (Upadhyay et al., 2021). In order to measure the five concepts of interest, we developed the survey using existing scales that operationalize the variables of interest (economic performance, environmental performance, operational performance, IoT based on I4.0, green design, recycling and remanufacturing, and green manufacturing). The data was gathered from Chinese food industries. To collect primary data, a structured questionnaire was used. A 5-point Likert scale was used to build the instrument. A multiple-item, 5-point Likert-type scale (1 = “Strongly Disagree”; 2 = “Disagree”; 3 = “Neutral”; 4 = “Agree”; 5 = “Strongly Agree”) was used. The study’s conceptual framework is depicted in Figure 1.

Researchers used data gathered from Chinese companies in the summer of 2019 to combine IoT based Industry 4.0 applications with CE principles to improve organizational performance. The primary goal of the survey is to look into how companies’ performance can be improved through the use of Industry 4.0 technologies and CE principles. The data gathering process was followed in the current investigation. Respondents’ socioeconomic status was shown in Table 2. We sent out 600 questionnaires throughout the summer of 2021. The total number of completed questionnaires received was 447, of which 38 were deemed insufficiently complete. In order to do further research, we incorporated all 409 remaining questions in our sample. Although the sample size was sufficient to use PLS-SEM to test hypotheses, only 67% of those who participated in the survey responded (Bag et al., 2021). Table 2 presents the demographic profile of the participants

**TABLE 2:** Demographic profile of the participants.

Characteristics	*N*	%
**Title**		
Vice president	14	3.42
General manager	54	13.20
Plant manager	64	15.65
Procurement manager	34	8.31
Logistics manager	65	15.89
Operation manager	82	20.05
Information system manager	96	23.47
**Work experience**		
<5	76	18.58
5–10	128	31.30
10–15	86	21.03
15–20	47	11.49
20–35	43	10.51
>35	29	7.09

### Data Analysis

The statistical software package SPSS 25 with AMOS 24 was used to analyze the respondents’ data. For SEM, Sharma et al. (2021) recommend a sample size of at least 200. Therefore, our sample of 409 exceeds the recommended value. Additionally, maximum likelihood estimation was used and is commonly used with sample sizes greater than 300 observations.

## Results Analysis and Findings

### Descriptive Statistics

Statistics such as average, variance, and coefficient of determination are shown in Table 3. After compiling demographic information, we tested the measurement model and survey instrument for convergent validity, reliability and discriminant validity. When looking for collinearity, variance inflation factors (VIFs) are employed. *F*-square values indicate the level of significance of each construct in the model by calculating the variance explained by each independent variable. When it came time to conduct SEM analysis, the hypothesized associations from the measurement model were re-sampled and examined. Based on the idea that the theoretical foundations of interrelated measures are statistically related, convergent validity is defined. The degree to which two variables should be correlated is known as convergent validity. Table 3 displayed the Cronbach’s alpha (CA), average extracted variance (AVE), and composite reliability (CR). Reliability and validity were found to be in line with established standards.

**TABLE 3:** Descriptive statistics of the data.

Variables	Observations	Mean	SD	Coefficient of variation (CV)
ECP	409	3.872	0.538	0.153
ENP	409	2.971	1.648	0.611
OP	409	3.534	0.267	0.084
GD	409	4.189	0.512	0.134
RR	409	2.851	0.605	0.233
GM	409	2.851	0.605	0.233
IoT	409	3.185	1.817	0.628

*IoT, Internet of Things; ENP, environmental performance; ECP, economic performance; OP, operational performance; CD, circular design; RR, recycling and remanufacturing; GM, green manufacturing.*

The Fornell and Larcher criterion is used to check discriminant validity. The results are presented in Table 4. The higher values in the diagonal indicate the discriminant validity. If the square root of the average variance extracted is greater than the square root of other bivariate relations, this specifies the discrimination is valid. Discriminant validity boosts the relationships between items and their respective constructs that are stronger than others.

**TABLE 4:** Results of discriminant validity.

Variable	IoT	GD	RR	GM	ECP	ENP	OP
IoT	0.8534						
GD	0.5722	0.7690					
RR	0.6672	0.6845	0.7565				
GM	0.6192	0.6240	0.6864	0.7987			
ECP	0.5779	0.6566	0.5866	0.5184	0.7315		
ENP	0.4080	0.7133	0.6432	0.6298	0.6240	0.8054	
OP	0.7190	0.6854	0.7027	0.5971	0.6163	0.7056	0.8266

### Exploratory Factor Analysis

Previous Industry 4.0 and supply chain management studies have employed the exploratory factor analysis (EFA) method. One of EFA’s primary functions is discovering the structural relationships among several variables. Table 5 shows that the KMO value is 0.87, which is higher than the 0.60 suggested minimum (Camana et al., 2021). The outcome of Bartlett’s Test of Sphericity was also relevant in light of the resources examined in this research. This means that EFA can be used with these resources. Table 6 depicts the rotated component matrix after it has been rotated. It reveals that 25 materials were categorized into seven different categories. Factor loadings exceeded 0.50% in all cases. The eigenvalues were all greater than or equal to 1.00.

**TABLE 5:** KMO and Bartlett’s Test.

Kaiser-Meyer-Olkin measure of sampling adequacy		0.864
Bartlett’s Test of Sphericity	5,264.465	5,264.465
	260.59	260.590
	0.000	0.000

**TABLE 6:** Cronbach’s alpha results.

Variables	Items	Standard loadings	Cronbach’s α	CR
IoT based on I4.0 (IoT)			0.903	0.925
	IoT1	0.634		
	IoT2	0.841		
	IoT3	0.802		
	IoT4	0.869		
Circular design (CD)			0.832	0.893
	CD1	0.851		
	CD2	0.736		
	CD3	0.661		
	CD4	0.914		
Remanufacturing and recycling (RR)			0.809	0.832
	RR1	0.746		
	RR2	0.71		
	RR3	0.762		
Green manufacturing (GRNM)			0.923	0.932
	GM1	0.837		
	GM2	0.801		
	GM3	0.853		
Economic performance			0.813	0.807
	ECP1	0.737		
	ECP2	0.802		
	ECP3	0.92		
	ECP4	0.866		
Environmental performance			0.916	0.935
	ENP1	0.719		
	ENP2	0.731		
	ENP3	0.731		
	ENP4	0.675		
Operational performance			0.91	0.915
	OP1	0.88		
	OP2	0.959		
	OP3	0.709		

### Multicollinearity Test

The VIF is a common measure used to assess the multicollinearity between independent variables. Belhadi et al. (2021) recommend a VIF score below 5.0 to demonstrate that multicollinearity is not an issue among the independent variables. Table 7 below lists the results of the multicollinearity test. The VIF scores range between 1.993 and 3.971 falling within the acceptable range for VIF scores.

**TABLE 7:** Multicollinearity test.

Variables	Unstandardized coefficients		Standardized coefficients			Collinearity statistics	
	*B*	SE	Beta	*t*	Sig.	Tolerance	VIF
(Constant)	0.066	0.199	0.000	0.347	0.789	0.000	0.000
IoT	0.142	0.051	0.142	2.930	0.006	0.564	1.993
GD	0.019	0.058	0.018	0.348	0.788	0.472	2.384
RR	0.046	0.052	0.054	0.933	0.402	0.399	2.821
GM	0.102	0.058	0.110	1.849	0.087	0.374	3.001
ECP	0.116	0.070	0.120	1.744	0.107	0.283	3.971
ENP	0.251	0.057	0.260	4.618	0.000	0.421	2.669
OP	0.244	0.061	0.237	4.170	0.000	0.409	2.748

### Common Method Bias

Harman’s single factor test was conducted to ensure that the model is free from common method bias. The SPSS software package was used to derive the result by conducting an un-rotated, single-factor constraint factor analysis. As demonstrated in Table 8, the highest variance explained by one factor was 47.225%. This is below the 50% cutoff point indicating that no concern with common method bias exists.

**TABLE 8:** Total variance explained.

	Initial eigenvalues			Extraction sums of squared loadings		
Components	Total	Variance %	Cumulative %	Total	Variance %	Cumulative %
1	13.460	44.864	44.864	13.460	44.864	44.864
2	2.630	8.765	53.628	2.630	8.765	53.628
3	1.202	4.006	57.635	1.202	4.006	57.635
4	1.022	3.409	61.043	1.022	3.409	61.043
5	0.851	2.837	63.880	0.851	2.837	63.880
6	0.783	2.611	66.491	0.783	2.611	66.491
7	0.687	2.290	68.780	0.687	2.290	68.780
8	0.624	2.081	70.861	0.624	2.081	70.861
9	0.603	2.012	72.874	0.603	2.012	72.874
10	0.532	1.775	74.648			
11	0.501	1.668	76.316			
12	0.485	1.619	77.935			
13	0.426	1.419	79.354			
14	0.400	1.332	80.686			
15	0.376	1.255	81.941			
16	0.367	1.223	83.164			
17	0.356	1.188	84.351			
18	0.345	1.150	85.501			
19	0.318	1.060	86.561			
20	0.300	0.999	87.562			
21	0.286	0.952	88.513			
22	0.271	0.902	89.415			
23	0.265	0.884	90.298			
24	0.239	0.799	91.097			
25	0.238	0.792	91.890			

### Analysis of Factor Loadings

Convergent validity is measured through the factor loadings and cross-loadings of the survey items. Table 9 lists the results of the factor analysis. Hair et al. (2012) recommend that the factor loadings should be at a level of 0.5 or greater. All survey items that fell below the recommended level were removed from the study. The remaining 23 survey items all met the acceptable level of validity and explained 76.709% of the variance in the dependent construct.

**TABLE 9:** Factor analysis.

Variable	IoT	CD	GM	RR	ENP	ECP	OP
IoT 1	**0.799**						
IoT 2	**0.788**						
IoT 3	**0.776**						
IoT 4	**0.690**						
CD 3		**0.784**					
CD 4		**0.783**					
CD 2		**0.782**					
GM 2			**0.795**				
GM 1			**0.787**				
GM 3			**0.784**				
RR_1				**0.826**			
RR_4				**0.826**			
RR_2				**0.826**			
RR_3				**0.826**			
ENP_2					**0.812**		
ENP_3					**0.796**		
ENP_1					**0.783**		
ECP 3						**0.832**	
ECP 4						**0.809**	
ECP 2						**0.804**	
ECP 1						**0.777**	
OP 1							**0.815**
OP 2							**0.803**
OP 3							**0.690**

*Bold figures depict variable cross-loadings.*

### Structural Equation Model

SPSS AMOS 24 was utilized to evaluate the proposed research model. To test the overall goodness of fit of the proposed research model, the measures of df/Chi-square, goodness of fit, adjusted goodness of fit, root mean square error of approximation, comparative fit index, Tucker Lewis index, and normed fit index were employed. Table 10 reveals that all the goodness of fit indices falls within their acceptable levels. This reveals that the proposed research model exhibited a good fit with the data.

**TABLE 10:** Fit indices for the models.

Indices of fit	Value recommended	Model value
df/Chi-square	≤3.00	1.293
Goodness of fit	≥0.90	0.995
Adjusted goodness of fit	≥0.80	0.959
Root mean square error of approximation	≤0.06	0.03
Comparative fit index	≥0.93	0.999
Tucker Lewis index	≥0.90	0.994
Normed fit index	≥0.90	0.996

### Hypothesis Testing

**FIGURE 2:**
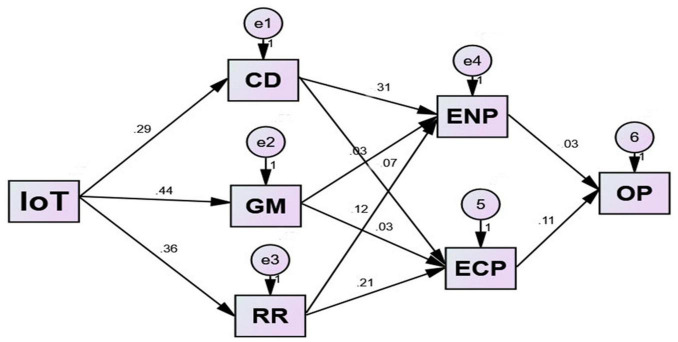
Path diagram.

Table 11 and Figure 2 below show the properties of the causal paths including the standardized path coefficients.

**TABLE 11:** The results of hypothesis testing.

Hypothesis	Hypothesis testing	β-Value	*f*-Value	Result
1	Internet of Things → circular design	0.295***	145.45	Accepted
2	Internet of Things → green manufacturing	0.447***	153.37	Accepted
3	Internet of Things → remanufacturing and recycling	0.364***	196.29	Accepted
4	Circular design → environmental performance	0.314**	235.82	Accepted
5	Green manufacturing → environmental performance	0.034***	182.64	Accepted
6	Remanufacturing and recycling → environmental performance	0.126***	227.56	Accepted
7	Circular design → economic performance	0.072**	139.44	Accepted
8	Green manufacturing → economic performance	0.286**	142.02	Accepted
9	Remanufacturing and recycling → economic performance	0.214***	151.89	Accepted
10	Economic performance → operational performance	0.036***	141.83	Accepted
11	Environmental performance → operational performance	0.114***	123.02	Accepted

*, **, *** means 10%, 5% and 1% significance level.

## Discussion

The findings show that all of the hypothesized relationships proposed in the model are significant. The IoT based Industry 4.0 technology has a substantial impact on the CE in all of its components, as may be deduced from Table 10. Circular design can be improved by a 0.295% increase in IoT usage for every one percent rise in IoT. Green manufacturing is also benefiting from IoT, as a 1% growth in the IoT would raise it by 0.447%. An increase of 1% in IoT’s impact on recycling and remanufacturing will result in an additional 0.364% increase in these processes. Researchers such as Chen et al. (2020) have also investigated this association in previous studies. To implement the CE effectively, it is important to focus on the technical aspects of this new paradigm, as large-scale automation and digitization have occurred in the fourth industrial revolution. The IoT, first presented by Flynn and Hacking (2019), has become a key component of Industry 4.0. There are numerous ways in which IoT might help the CE: it is transparent, decentralized, peer-to-peer data synchronization, smart contracting, and data immutability. Monitoring apparent environmental and social circumstances that might be causing health, environmental, and safety hazards is an essential application focus for the BCT, like Adams et al. (2018) claim. Circular economies have a good impact on both the environment and the economy, and this influence is only growing. A total of 0.292, 0.215, and 0.262% of environmental performance are influenced by eco-friendly design, eco-friendly manufacturing, and recycling and remanufacturing. Ymeri et al. (2020) found that pollution in the environment is directly linked to the use of fossil fuels and energy in the production and distribution of goods. Additional emphasis was placed on green manufacturing and environmentally friendly transportation systems to reduce their negative impact on the environment and society. As stated by Soo et al. (2021), the CE or GSCM improves resource efficiency and safeguards the environment. Circular economies have been proposed by Han and Trimi (2022), and this is one of their central claims, which they analyze further in this research by looking at the effect that environmental performance and economic performance have on organizational performance.

Eco-friendly design and manufacturing contribute 0.314, 0.034, and 0.126% to the economy’s performance, respectively. Remanufacturing and recycling goods can save billions of dollars each year, according to Ghadimi et al. (2019). Most organizations, according to Çalık (2021), only engage in green activities that increase their profits and help them enter the export market. The results show that a 1% increase in environmental performance might result in a 0.114% increase in operational performance, proving the link between environmental and economic performance. Kolla et al. (2019) investigated the data obtained from Chinese enterprises to evaluate the effectiveness of activities related to GSCM. They concluded that the organizations’ goals and profitability are boosted by sustainability. As a result, environmental and economic performance improve the company’s performance by 0.036 and 0.114%, respectively. Kumar et al. (2021) observed that green practices have the potential to improve a company’s overall performance, but the lack of support from high management can quickly result in a disastrous GSCM project. Gupta et al. (2021) found that economic considerations have an impact on an organization’s performance.

## Conclusion

The IoT and the CE are two hot issues that will have a significant impact on business and society. One of the first studies to look at how IoT can help the CE. Industrial case studies are used to examine potential and identify application gaps for future research and practice.

They are all representative of their respective industries. All of the cases have claimed the benefits of IoT for their supply chains and the CE in general. Adoption has been mainly at the demonstration and piloting stages so far, nevertheless, Interoperability problems, security concerns, and stability issues with IoT systems are still present. Even though industrial trends remain good and preliminary results show that industry leaders such as Walmart and Toyota mimic one another’s behavior, the majority of smaller businesses are skeptical and reluctant to embrace this technological transformation. As a result of these tensions, there is a risk of increased overhead, technological investment, and a lack of immediate financial rewards. Small, risk-taking businesses are more likely to adopt new technologies than larger ones.

With IoT, businesses may expect a more transparent, decentralized, and secure transaction process as well as the potential for increased efficiency and responsiveness. In the long run, these attributes will have a positive impact on the economy, society, and the environment.

Decentralization and digitalization can help the CE by improving supply chain procedures in each industrial sector that are lacking in transparency, precision, and security – all of which can benefit from decentralization and digitalization. The adoption of IoT for various CE procedures varies significantly among industries, as we have seen. The case studies that have been provided allow us to understand how many firms from various industries have progressed toward the use of IoT. CE implementation dimensions and their case scenarios can also be viewed in this way. This study’s findings may help clarify how IoT can be used in cases of CE. Additionally, managers, policymakers, and CE coordinators can use real illustrative instances to identify the practical gaps that organizations can focus on when they attempt to use their CE practices with IoT.

An examination into whether IoT-based solutions may benefit businesses and industries in the CE is needed. A good place to start researching industries and organizations is by identifying the most critical operational principles and sector-specific difficulties that IoTs and CE are currently confronted with. Legal framework and relationship requirements, application scale, and infrastructure needs can all be included in this list of principles. Further investigation should be done into the supply chain activities and features that can be improved by a IoT-powered solution. In a IoT ecosystem, procurement, shipping, and logistics may have a higher impact on CE than internal processes. IoT may not be necessary for every situation, and basic or even classic digital solutions may suffice in many scenarios.

Our exploratory inquiry has reached its limits. In order to support a large-scale investment in IoT and CE links, the data from the case studies is insufficient. There is a need for more evidence. It is essential to include business cases and long-term observations into IoT in the CE environment in order to maximize its efficacy. IoT’s role in today’s supply chains is defined by the process of creating, implementing, and enforcing essential IoT characteristics.

The IoT offers a wide range of applications, each of which requires a large amount of infrastructure. So, it is with the methods of continuing education. Both fields have a large number of potential applications and connections. In this relationship, there are both opportunities and challenges. Because significant sectors have already embraced and implemented the technology at such a rapid pace, more concrete evidence is needed. It is also necessary to conduct a more rigorous analysis of the potential of IoT in a CE environment. That is why we believe that our work sets the groundwork for major advancement in both domains.

## Data Availability Statement

The raw data supporting the conclusions of this article will be made available by the authors, without undue reservation.

## Author Contributions

Both authors listed have made a substantial, direct, and intellectual contribution to the work, and approved it for publication.

## Conflict of Interest

The authors declare that the research was conducted in the absence of any commercial or financial relationships that could be construed as a potential conflict of interest.

## Publisher’s Note

All claims expressed in this article are solely those of the authors and do not necessarily represent those of their affiliated organizations, or those of the publisher, the editors and the reviewers. Any product that may be evaluated in this article, or claim that may be made by its manufacturer, is not guaranteed or endorsed by the publisher.
